# Deep learning assessment of disproportionately enlarged subarachnoid-space hydrocephalus in Hakim’s disease or idiopathic normal pressure hydrocephalus

**DOI:** 10.1093/radadv/umae027

**Published:** 2024-11-04

**Authors:** Shigeki Yamada, Hirotaka Ito, Chifumi Iseki, Toshiyuki Kondo, Tomoyasu Yamanaka, Motoki Tanikawa, Tomohiro Otani, Satoshi Ii, Yasuyuki Ohta, Yoshiyuki Watanabe, Shigeo Wada, Marie Oshima, Mitsuhito Mase

**Affiliations:** Department of Neurosurgery, Nagoya City University Graduate School of Medical Science, Nagoya, Aichi, 467-8601, Japan; Interfaculty Initiative in Information Studies/Institute of Industrial Science, The University of Tokyo, Tokyo, 113-8654, Japan; Medical System Research & Development Center, FUJIFILM Corporation, Tokyo, 107-0052, Japan; Department of Behavioral Neurology and Cognitive Neuroscience, Tohoku University Graduate School of Medicine, Sendai, Miyagi, 980-8575, Japan; Division of Neurology and Clinical Neuroscience, Department of Internal Medicine III, Yamagata University School of Medicine, Yamagata, Yamagata, 990-2331, Japan; Division of Neurology and Clinical Neuroscience, Department of Internal Medicine III, Yamagata University School of Medicine, Yamagata, Yamagata, 990-2331, Japan; Department of Neurosurgery, Nagoya City University Graduate School of Medical Science, Nagoya, Aichi, 467-8601, Japan; Department of Neurosurgery, Nagoya City University Graduate School of Medical Science, Nagoya, Aichi, 467-8601, Japan; Department of Mechanical Science and Bioengineering, Graduate School of Engineering Science, Osaka University, Toyonaka, Osaka, 560-8531, Japan; Department of Mechanical Engineering, School of Engineering, Institute of Science Tokyo, Tokyo, 152-8550, Japan; Division of Neurology and Clinical Neuroscience, Department of Internal Medicine III, Yamagata University School of Medicine, Yamagata, Yamagata, 990-2331, Japan; Department of Radiology, Shiga University of Medical Science, Otsu, Shiga, 520-2192, Japan; Division of Neurology and Clinical Neuroscience, Department of Internal Medicine III, Yamagata University School of Medicine, Yamagata, Yamagata, 990-2331, Japan; Interfaculty Initiative in Information Studies/Institute of Industrial Science, The University of Tokyo, Tokyo, 113-8654, Japan; Department of Neurosurgery, Nagoya City University Graduate School of Medical Science, Nagoya, Aichi, 467-8601, Japan

**Keywords:** MRI, disproportionately enlarged subarachnoid-space hydrocephalus, DESH, Hakim’s disease, idiopathic normal pressure hydrocephalus, iNPH, chronic hydrocephalus in adults, artificial intelligence, automatic segmentation

## Abstract

**Background:**

Disproportionately enlarged subarachnoid-space hydrocephalus (DESH) is a key feature of Hakim’s disease (synonymous with idiopathic normal pressure hydrocephalus; iNPH). However, it previously had been only subjectively evaluated.

**Purpose:**

This study aims to evaluate the usefulness of MRI indices, derived from deep learning segmentation of cerebrospinal fluid (CSF) spaces, for DESH detection and to establish their optimal thresholds.

**Materials and Methods:**

This study retrospectively enrolled a total of 1009 participants, including 77 patients diagnosed with Hakim’s disease, 380 healthy volunteers, 163 with mild cognitive impairment, 256 with Alzheimer’s disease, and 217 with other types of neurodegenerative diseases. DESH, ventriculomegaly, tightened sulci in the high convexities, and Sylvian fissure dilatation were evaluated on three-dimensional T1-weighted MRI by radiologists. The total ventricles, high-convexity part of the subarachnoid space, and Sylvian fissure and basal cistern were automatically segmented using the CSF Space Analysis application (FUJIFILM Corporation). Moreover, DESH, Venthi, and Sylhi indices were calculated based on these 3 regions. The area under the receiver-operating characteristic curves of these indices and region volumes (volume ratios) for DESH detection were calculated.

**Results:**

Of the 1009 participants, 101 (10%) presented with DESH. The DESH, Venthi, and Sylhi indices performed well with 95.0%-96.0% sensitivity and 91.5%-96.8% specificity at optimal thresholds. All patients with Hakim’s disease were diagnosed with DESH, despite variations in severity. In patients with Hakim’s disease, with or without Alzheimer’s disease, the DESH index and total ventricular volume were significantly higher compared to patients with Alzheimer’s disease, although the total intracranial cerebrospinal fluid volume was significantly lower.

**Conclusion:**

DESH, Venthi, and Sylhi indices, and the volumes and volume ratios of the ventricle and high-convexity part of the subarachnoid space computed using deep learning were useful for the DESH detection that may help to improve the diagnosis of Hakim’s disease (ie, iNPH).

**Abbreviations**

AVIM = asymptomatic ventriculomegaly with features of iNPH on MRI; CHiA = Chronic hydrocephalus in adults; CSF = cerebrospinal fluid; DESH = Disproportionately enlarged subarachnoid-space hydrocephalus; iNPH = idiopathic normal pressure hydrocephalus; MCI = mild cognitive impairment; MMSE = mini-mental state examination; ROIs = regions of interest; SFD = Sylvian fissure dilation; THC = tightened sulci in the high convexities.

**Summary**

Disproportionately enlarged subarachnoid-space hydrocephalus (DESH), the most useful feature for detecting Hakim’s disease (ie, idiopathic normal pressure hydrocephalus), is quantitated through deep learning-based extraction of 3 specific regions on MRI.

**Key Results**

- Quantitative evaluation of disproportionately enlarged subarachnoid-space hydrocephalus (DESH) in Hakim’s disease, that is, idiopathic normal pressure hydrocephalus, is achieved with deep learning segmentation of the cerebrospinal fluid on MRI.
- DESH, Venthi, and Sylhi indices enabled diagnosis of DESH in clinically suspected Hakim’s disease with 95.0%-96.0% sensitivity and 91.5%-96.8% specificity.
- DESH index is strongly influenced by the volume of the tightened sulci in the high convexities, with smaller volumes having the greatest impact.

## Introduction

Chronic hydrocephalus in adults (CHiA), which had been called “normal pressure hydrocephalus (NPH)” is classified into idiopathic (iNPH) and secondary NPH.^1^^,^^2^ Secondary NPH develops after subarachnoid hemorrhage, head injury, brain tumors, or other preceding conditions.^3–5^ This classification was first proposed by Hakim and Adams et al. in 1965^6^ and remains in use today. In 2024, the International Society for Hydrocephalus and Cerebrospinal Fluid Disorders (ISHCSF) task force team proposed a new classification of 7 categories defined by age and clinical parameters: (1) Hakim’s disease (classically iNPH), (2) early midlife hydrocephalus, (3) late midlife hydrocephalus, (4) secondary hydrocephalus, (5) compensated hydrocephalus, (6) genetic hydrocephalus, and (7) transitioned hydrocephalus.^7^

Recently, Hakim’s disease (iNPH) has been recognized as a common disease among the elderly, with a large proportion in aged societies, such as Japan and South Korea. According to previous epidemiological studies,^8–13^ the probability of patients with Hakim’s disease (iNPH) receiving appropriate diagnosis and treatments has been estimated to be less than 10% of all potential patients, indicating large regional differences. Hakim’s triad symptoms—gait disturbance, cognitive impairment, and incontinence—are progressive, with patients becoming less independent and requiring nursing care,^2^^,^^14–17^ eventually leading to death.^18^^,^^19^

Hakim’s disease (iNPH) is still often undetected or misdiagnosed as ventriculomegaly is less prominent in some cases. Doctors unfamiliar with Hakim’s disease (iNPH) may mistake the concurrent enlargement of ventricles and the Sylvian fissure for focal brain atrophy, leading to a misdiagnosis of Alzheimer’s disease. To distinguish Hakim’s disease from Alzheimer’s disease, disproportionately enlarged subarachnoid-space hydrocephalus (DESH),^20–22^ including tightened sulci in the high convexities (THC),^23–28^ has been recently considered as the most important imaging feature specific to Hakim’s disease. DESH refers to the unbalanced distribution of cerebrospinal fluid (CSF), that is, the simultaneous occurrence of ventriculomegaly, Sylvian fissure dilation (SFD), and THC. Although DESH has been increasingly recognized as a neuroimaging hallmark of Hakim’s disease, subjective evaluation of DESH remains ambiguous and inconsistent among experts.^21^^,^^24^^,^^26^^,^^27^

We recently developed a deep learning model to automatically extract the regions of interest (ROIs) for DESH assessment, including ventriculomegaly, SFD, and THC from three-dimensional (3D) MRIs.^29^ In addition, we proposed several indices calculated from the segmented ROIs for DESH assessment.^29^ This study aims to evaluate the usefulness of these indices for DESH detection as well as the volumes and volume ratios of ROIs with a newly released deep learning-based automatic CSF segmentation application using a large 3D MRI dataset from multicenter collaborative studies. Furthermore, we aimed to establish thresholds for the indices and volumes and volume ratios of the ROIs for quantitative evaluation of DESH to improve radiological evaluation of Hakim’s disease (iNPH).

## Materials and methods

### Ethical approvals

The study design and protocol of this prospective and observational study were approved by the ethics committees for human research at our institutes (IRB number: 60-22-0083, R2019-227).

### Study population

The dataset of 3D T1-weighted MRIs in this study included patients from the 3 collaborating hospitals and volunteers from 2 cohorts, with data collected retrospectively and continuously. One cohort comprised of volunteers aged 60 years or older who participated in the Takahata population-based cohort study,^11^ whereas another cohort had healthy volunteers aged ≥20 years recruited from among medical staff, students, and their families by open recruitment between November 2020 and February 2022.^30^^,^^31^ The inclusion criteria defined healthy subjects as individuals with no previous history of brain injury, brain tumor, or cerebrovascular disease on brain MRI examinations and who had no neurological symptoms, including compromised cognitive function. The inclusion criteria for patients were individuals with suspected brain disorders who had not undergone brain surgery and had undergone a 3D T1-weighted MRI. The criteria for Hakim’s disease (iNPH) included the presence of DESH and at least 1 of Hakim’s triad symptoms according to the third edition of the Japanese guidelines for the management of iNPH.^2^ Neurodegenerative diseases, including Alzheimer’s disease and Parkinson’s disease, were diagnosed by neurologists. The exclusion criteria were as follows: CHiA in 6 categories except for Hakim’s disease, the presence of space-occupying lesions in the brain, or imaging data that cannot be processed using the CSF Space Analysis and Brain Subregion Analysis applications on an independent 3D volume analyzer workstation (SYNAPSE 3D; FUJIFILM Corporation). Nine were excluded for not working on these applications. Three MRI data were excluded because 2 were incidentally found to have chronic subdural hematomas and 1 was found to have a brain tumor. A total of 1009 subjects were included in this study, with 517 of the 1009 participants having been previously reported.^4^^,^^5^^,^^11^^,^^28–35^ One prior article dealt with the prevalence of “asymptomatic ventriculomegaly with features of iNPH on MRI” (AVIM)^10^ and Hakim’s disease in the rural community.^11^ The other articles dealt with CSF distribution pattern in Hakim’s disease and other diseases,^4^^,^^5^^,^^32^^,^^33^ changing CSF distribution after shunt surgery in Hakim’s disease,^34^ CSF dynamics in Hakim’s disease on four-dimensional flow imaging,^35^ the volumes and volume ratios of segmented brain and CSF in healthy population,^30^ quantification of the CSF motion by using intravoxel incoherent motion MRI,^31^ definition of THC used in this study,^28^ and development of automatic quantitative assessment of DESH.^29^ In all previous studies, DESH has been evaluated subjectively. The current study is the first application-based quantitative evaluation of DESH.

### Image acquisitions

**Figure 1.**
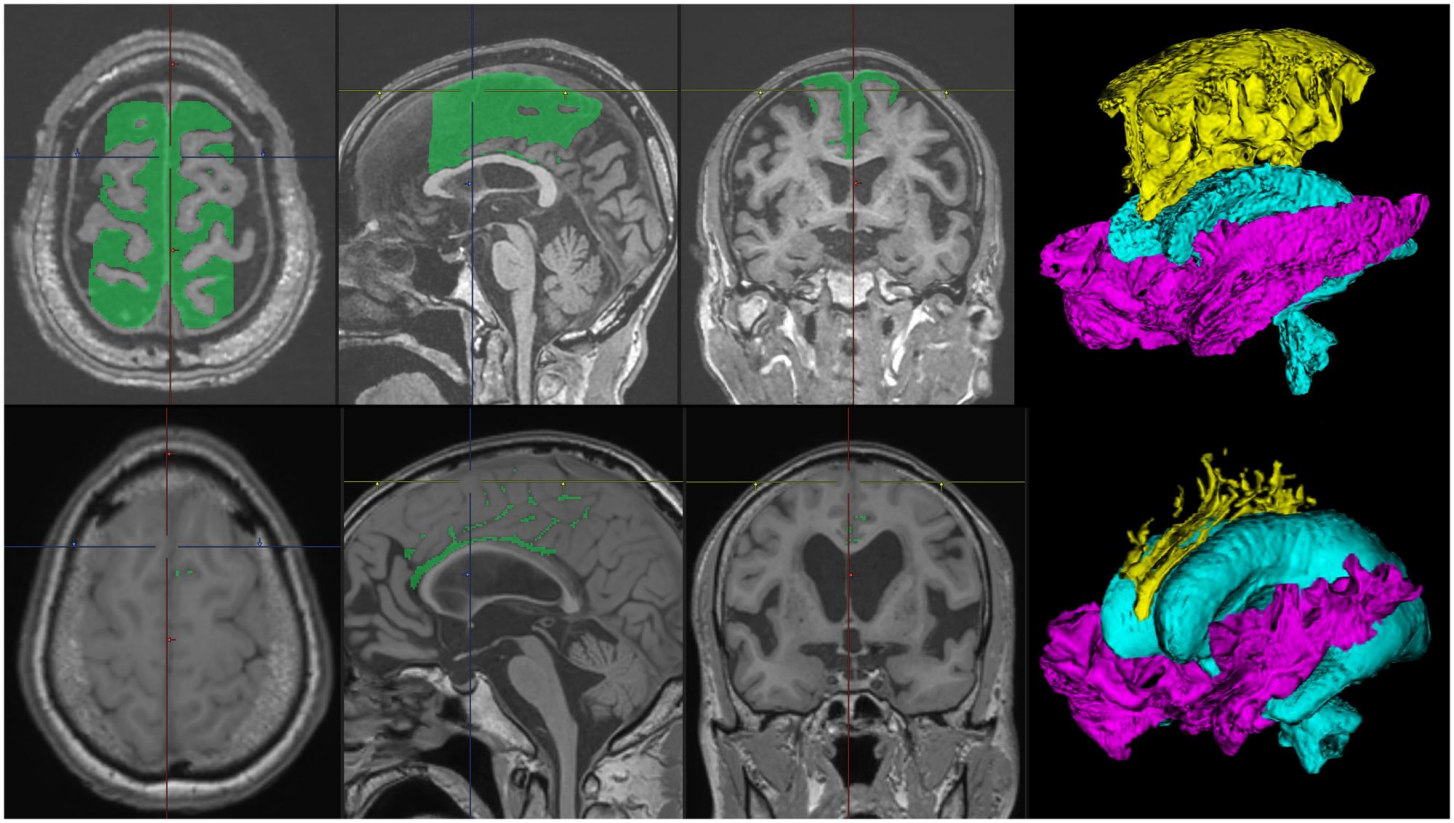
Segmentation of disproportionately enlarged subarachnoid space hydrocephalus (DESH) from 3D T1-weighted MRI in Hakim’s disease (idiopathic normal pressure hydrocephalus, iNPH). The cerebrospinal fluid (CSF) Space Analysis application on the 3D volume analyzer SYNAPSE 3D workstation (FUJIFILM Corporation) quickly and automatically segmented the 3 following regions of interest: the total ventricles (sky blue), high-convexity part of the subarachnoid space, and Sylvian fissure and basal cistern in the right figures. The highlighted regions in the left triaxial sectional views indicate the high-convexity part of the subarachnoid space. The upper figures show the brain of an elderly healthy volunteer with brain atrophy, whereas the lower figures show the brain of a patient with Hakim’s disease (iNPH) with a typical DESH feature.

**Figure 2.**
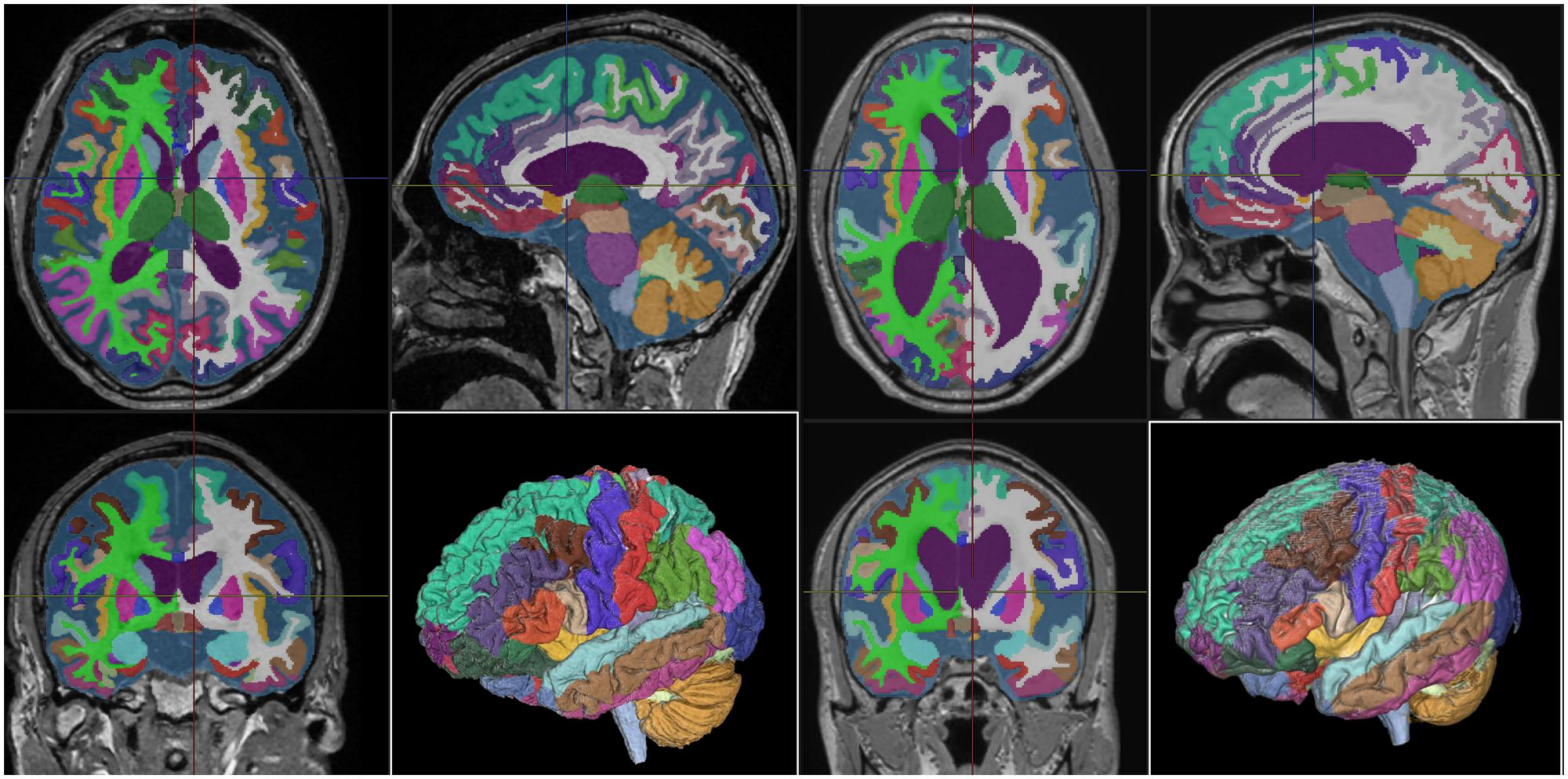
Brain and cerebrospinal fluid (CSF) segmentation from three-dimensional (3D) T1-weighted MRI. The brain subregion analysis application on the 3D volume analyzer SYNAPSE 3D workstation (FUJIFILM Corporation) quickly and automatically segmented the intracranial space into the 100 brain subregions, 4 ventricles, and 1 subarachnoid space from the 3D T1-weighted MRI images. The upper figures show the brain of an elderly healthy volunteer with brain atrophy, whereas the lower figures show the brain of a patient with Hakim’s disease (idiopathic normal pressure hydrocephalus) with a typical disproportionately enlarged subarachnoid space hydrocephalus (DESH) feature.

Based on the 3D T1-weighted MRI, the total ventricles, high-convexity part of the subarachnoid space, and Sylvian fissure and basal cistern were automatically segmented using the CSF Space Analysis application (Figure 1),^29^ while the intracranial space and total CSF space including the ventricles and subarachnoid spaces were also automatically segmented using the Brain Subregion Analysis application (Figure 2).^30^ All the 3D T1-weighted MRIs were magnetization-prepared rapid gradient echo sequences on 1.5-tesla or 3-tesla MRI machines made by GE Healthcare (United States), Siemens AG (Germany), and Philips (Netherlands) at the collaborating hospitals. The CSF Space Analysis application checks the 2D and 3D images to determine whether the 3 ROIs have been extracted accurately and performs corrections to the ROIs manually. The high-convexity part of the subarachnoid space was defined as the location above the body of the lateral ventricles, with the lateral end 3 cm from the midline, posterior end in the bilateral posterior parts of the callosomarginal sulci, and anterior end on the coronal plane perpendicular to the anterior commissure–posterior commissure line passing through the front edge of the genu of the corpus callosum.^28^

### 3D volumetric index

The “DESH index” was defined as the combined volume of the total ventricles and Sylvian fissure and basal cistern divided by the high-convexity part of the subarachnoid space volume.^29^ In relation to the supplemental indices for DESH, the “Venthi index” was defined as the total ventricular volume divided by the high-convexity part of the subarachnoid space volume, while the “Sylhi index” was defined as the volume of the Sylvian fissure and basal cistern divided by the high-convexity part of the subarachnoid space volume. These 3 indices were automatically calculated by the segmented volumes on the CSF Space Analysis application.

### Radiologist image analysis

The presence or absence of DESH, ventriculomegaly, THC, and SFD was evaluated subjectively by 2 experts in a blinded fashion, 1 certified radiologist from each institute taken on MRI and 1 being the leader of this study. If the 2 judges differed, the final decision was made by a third adjudicating radiologist.

### Statistical analyses

The mean and standard deviation of age, mini-mental state examination, indices, segmented volumes, and volume ratios, which were defined as the segmented volumes divided by the intracranial space were compared between the DESH and non-DESH groups using the Mann–Whitney–Wilcoxon test. The proportions of the 2 groups were compared using the Chi-square test. The distribution of the automatically segmented volumes, volume ratios, and indices was examined using fitting curves with nonlinear regression analysis. The area under the receiver operating characteristic curves (AUCs) and optimal thresholds for detecting DESH, ventriculomegaly, THC, and SFD were calculated to maximize the sum of the sensitivities and specificities by maximizing Youden’s index. Using the optimal thresholds from AUC analyses, the odds ratios (ORs) with 95% confidential intervals using thresholds were calculated. All missing variables were considered as deficit data, and no other variables were adjusted. Statistical significance was set at a probability value (*P*) of <0.001. All statistical analyses were performed using the R software (version 4.4.0, R Foundation for Statistical Computing, Vienna, Austria, http://www.R-project.org).

## Results

### Clinical characteristics

We enrolled a total of 1009 participants from 3 collaborating hospitals and 2 cohorts in a retrospective, consecutive case manner. The participants comprised 77 patients diagnosed with Hakim’s disease (iNPH); 256 with Alzheimer’s disease; 24 with Lewy body dementia; 7 with frontotemporal dementia; 81 with mixed dementia; 6 with movement disorders without dementia, including Parkinson’s disease and corticobasal degeneration; 10 with psychiatric disorders; 5 with stroke; 163 with mild cognitive impairment (MCI); and 380 healthy volunteers (Table 1).

**Table 1.** Clinical characteristics of the study population.

	Total	DESH	Non-DESH	*P*
Total	1009	101 (10%)	908 (90%)	
Female	587	43 (7%)	544 (93%)	<.001
Male	422	58 (14%)	364 (86%)	
<60 years	109	1 (%)	108 (99%)	<.001
60-69 years	83	8 (10%)	75 (90%)	
70-79 years	488	49 (10%)	439 (90%)	
80 years or older	329	43 (13%)	286 (87%)	
Hakim’s disease (ie, idiopathic normal pressure hydrocephalus)	52	52 (100%)	0	<.001
Hakim’s disease with Alzheimer’s disease	25	25 (100%)	0	
Alzheimer’s disease	256	3 (1%)	253 (99%)	
Normal	380	12 (3%)	368 (97%)	
Mild cognitive impairment	163	5 (3%)	158 (97%)	
Mixed dementia	81	2 (2%)	79 (98%)	
Dementia with Lewy bodies	24	0	24 (100%)	
Frontotemporal dementia	7	0	7 (100%)	
Movement disorders without dementia	6	0	6 (100%)	
Psychiatric disorders	10	1 (10%)	9 (90%)	
Stroke	5	1 (20%)	4 (80%)	
Ventriculomegaly	195	97 (50%)	98 (50%)	<.001
THC	93	92 (99%)	1 (1%)	<.001
SFD	241	81 (34%)	160 (66%)	<.001

*P*, probability values between the DESH (disproportionately enlarged subarachnoid space hydrocephalus) and non-DESH group were calculated by the Mann–Whitney–Wilcoxon test or Chi-square test.

Abbreviations: SFD = Sylvian fissure dilation; THC = tightened sulci in the high convexities.

Of the 77 patients with Hakim’s disease, 25 had Alzheimer’s disease. Overall, 56 Hakim’s patients (73%) underwent the CSF tap test, while 41 (53%) underwent CSF shunt surgery, and their symptoms improved by ≥1 point based on the results of the Modified Rankin Scale and/or the Japanese iNPH Grading Scale.^2^ Since some patients and normal volunteers were identified as having DESH but did not present with any typical Hakim’s triad symptoms of gait disturbance, cognitive impairment, or urinary incontinence, they were diagnosed with AVIM^10^ and were not diagnosed with Hakim’s disease. In total, 101 participants (10%) were determined to have DESH, while the remaining 908 (90%) were not. Of the 195 participants with ventriculomegaly, 50% had DESH and 50% were not. Compared with the non-DESH group, the DESH group had more than twice the mean volume and volume ratio of the total ventricles and less than one-half the mean volume and volume ratio of the high-convexity part of the subarachnoid space (Table S1). The volume and volume ratio of the Sylvian fissure and basal cistern were significantly different by about 1.2 times larger in the DESH group, while the total CSF volume was not different between the 2 groups.

### Relationship between the DESH index and volumes and volume ratios

**Figure 3.**
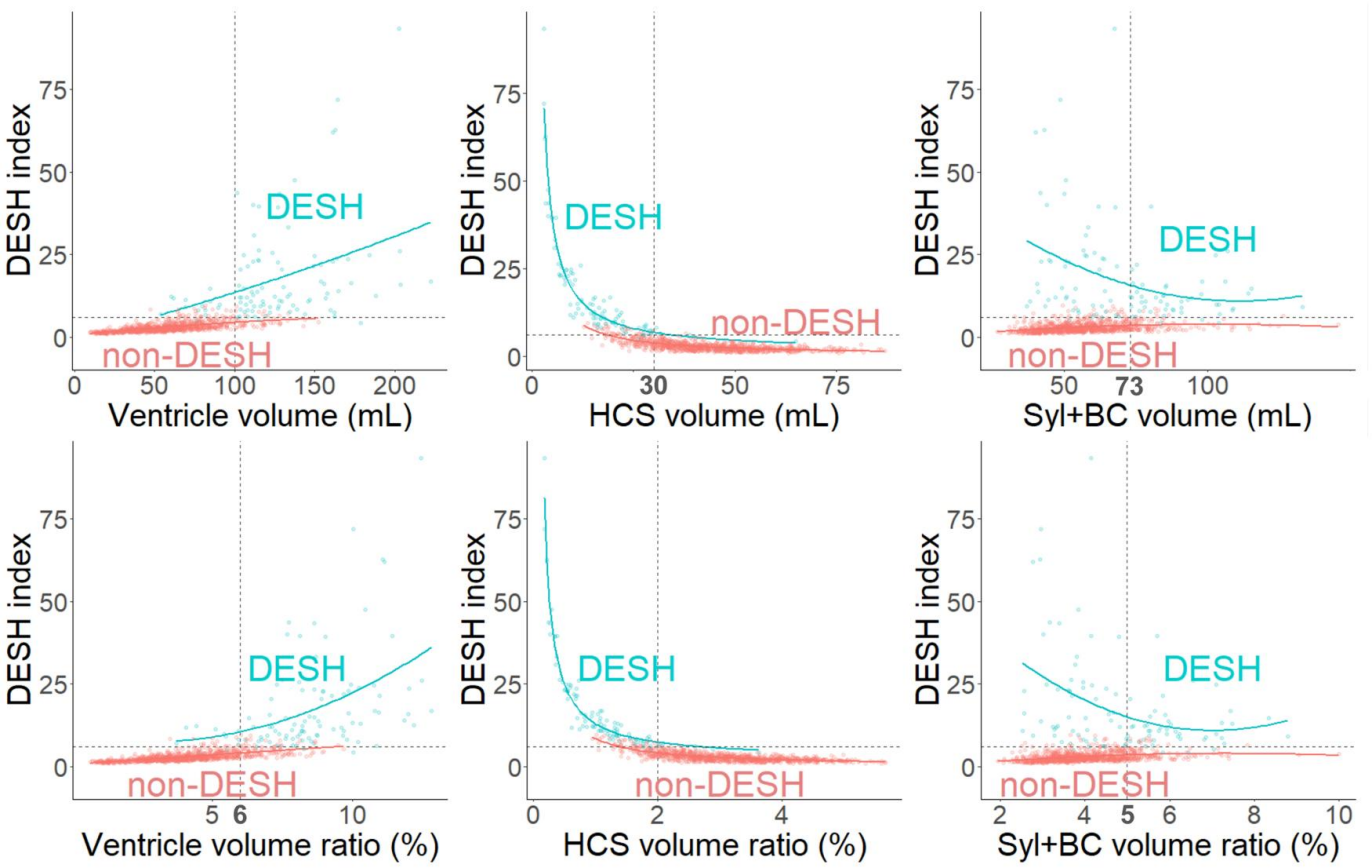
Distribution of the volumes and volume ratios of the 3 regions and disproportionately enlarged subarachnoid space hydrocephalus (DESH) index in the DESH and non-DESH groups. The scatter plots show the volumes (mL) and volume ratios (%) of the total ventricles, high-convexity part of the subarachnoid space, and Sylvian fissure and basal cistern (x-axis) and DESH index (y-axis). The lines indicate the fitting curves from the nonlinear regression analysis. HCS, high-convexity part of the subarachnoid space; Syl + BC, Sylvian fissure and basal cistern.

Figure 3 shows the relationship between the DESH index and the volumes and volume ratios of the 3 regions (total ventricles, high-convexity part of the subarachnoid space, and Sylvian fissure and basal cistern). Their distributions and fitting curves suggest that the DESH index had a linear relationship with the volume and volume ratio of the ventricles, whereas they were inversely proportional to the volume and volume ratio of the high-convexity part of the subarachnoid space, revealing a large contribution to the DESH index at small high-convexity subarachnoid space volumes and volume ratios.

### Comparison of extracted volumes, volume ratios, and indices in Hakim’s disease, Alzheimer’s disease, and their combination

All patients with Hakim’s disease were diagnosed with DESH, despite variations in severity of DESH (Table 2). Furthermore, in the Hakim’s disease and Hakim’s disease with Alzheimer’s disease groups, the ventricle volume and its volume ratio were significantly larger, while the volume ratio of the high-convexity subarachnoid space was significantly smaller compared to healthy volunteers, MCI, and Alzheimer’s disease groups. Additionally, the DESH index, Venthi index, and Sylhi index were all markedly elevated in the Hakim’s disease and Hakim’s disease with Alzheimer’s disease groups (*P *<* *0.001). In contrast, the total intracranial CSF volume was highest in the Alzheimer’s disease group, followed by the Hakim’s disease with Alzheimer’s disease group, which was significantly larger than in the Hakim’s disease group (*P *<* *0.001).

**Table 2.** Comparison of brain MRI features among Hakim’s disease (HD) (iodiopathic normal pressure hydrocephalus), Alzheimer’s disease (AD), HD with AD, Mild cognitive impairment (MCI), and normal volunteers (normal).

	HD	HD with AD	AD	MCI	Normal
Total	52	25	256	163	380
Female, *n* (%)	24 (46.2%)	10 (40%)	169 (66%)	82 (50.3%)	225 (59.2%)
DESH, *n* (%)	52 (100%)	25 (100%)	3 (1.2%)	5 (3.1%)	12 (3.2%)
Ventriculomegaly, *n* (%)	51 (98%)	25 (100%)	45 (18%)	17 (10%)	24 (6%)
THC, *n* (%)	50 (96.2%)	24 (96%)	3 (1.2%)	2 (1.2%)	12 (3.2%)
SFD, *n* (%)	39 (75%)	24 (96%)	64 (25%)	31 (19%)	40 (10.5%)
Age, mean	76.6 ± 7.2	81.4 ± 7.2	79.4 ± 17.4	77.6 ± 6.0	66.1 ± 17.4
Ventricle volume (mL), mean	134.7 ± 33.0	123.5 ± 30.8	71.0 ± 22.9	59.1 ± 20.5	45.0 ± 22.9
Ventricle volume ratio (%), mean	9.0 ± 1.8	8.5 ± 1.7	5.0 ± 1.5	4.1 ± 1.3	3.1 ± 1.5
HCS volume (mL), mean	14.4 ± 8.3	16.4 ± 7.0	48.7 ± 13.6	40.0 ± 14.3	40.0 ± 13.6
HCS volume ratio (%), mean	1.0 ± 0.6	1.1 ± 0.5	3.5 ± 0.9	2.8 ± 1.0	2.8 ± 0.9
Syl + BC volume (mL), mean	68.0 ± 20.1	80.6 ± 23.7	64.8 ± 11.9	54.8 ± 12.5	50 ± 11.9
Syl + BC volume ratio (%), mean	4.6 ± 1.3	5.6 ± 1.6	4.6 ± 0.7	3.8 ± 0.8	3.5 ± 0.7
CSF, mean	333.8 ± 43.5	381.9 ± 71.4	409 ± 62.4	332.9 ± 73.2	304.1 ± 62.4
DESH index, mean	**21.1 ± 17.1**	**17.1 ± 13.9**	3.1 ± 2.7	3.3 ± 2.0	2.9 ± 2.7
Venthi index, mean	**14.6 ± 13.2**	**11.2 ± 11.1**	1.6 ± 1.8	1.8 ± 1.3	1.5 ± 1.8
Sylhi index, mean	**6.5 ± 4.2**	**5.9 ± 3.1**	1.4 ± 1.0	1.5 ± 0.8	1.5 ± 1.0

DESH index = total ventricular volume, and the Sylvian fissure and basal cistern volume divided by the high-convexity part of the subarachnoid space volume; Venthi index = ventricular volume divided by the high-convexity part of the subarachnoid space volume; Sylhi index = Sylvian fissure and basal cistern volume divided by the high-convexity part of the subarachnoid space volume. The values shown in bold were significantly and markedly higher compared to the AD, MCI, and Normal groups.

Abbreviations: CSF = cerebrospinal fluid; DESH = disproportionately enlarged subarachnoid space hydrocephalus; HCS = high-convexity part of the subarachnoid space;SFD = Sylvian fissure dilation; THC = tightened sulci in the high convexities.

### Volumes and indices for DESH detection

**Figure 4.**
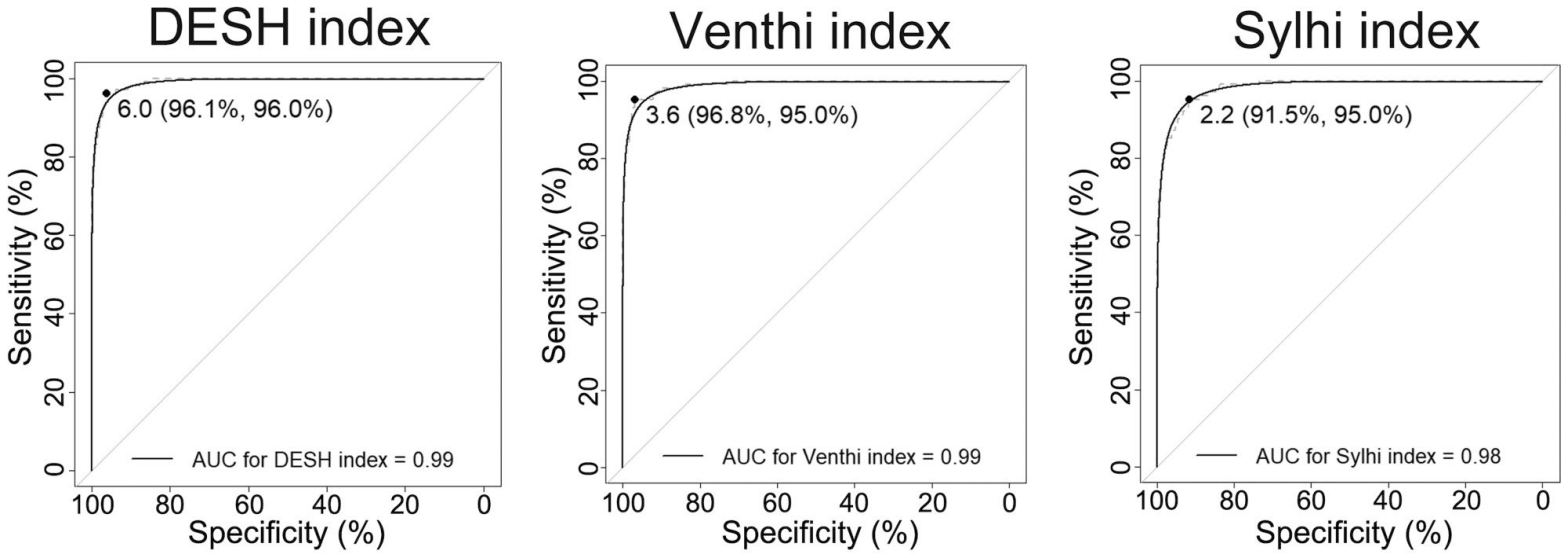
Receiver-operating characteristic (ROC) curves for DESH detection. The half-transparent lines indicate the fitted smooth binormal curves. The candidates for the optimal thresholds (sensitivities, specificities) are marked at the black points. DESH index = (total ventricle volume) + (Sylvian fissure and basal cistern volume)/(high-convexity part of the subarachnoid space volume). Venthi index = (total ventricle volume)/(high-convexity part of the subarachnoid space volume). Sylhi index = (Sylvian fissure and basal cistern volume)/(high-convexity part of the subarachnoid space volume).

The AUC of the DESH index for DESH detection was 0.99 (sensitivity, 96.0%; specificity, 96.1%) when the optimal threshold was set at 6 (Figure 4). Specifically, MRI with 6 or higher of the DESH index had a 468-fold higher OR to be DESH (Table 3). The AUCs of the Venthi and Sylhi indices for DESH detection were also sufficiently high (0.99 and 0.98, respectively). The AUCs of the simple volumes and volume ratios of total ventricles and high-convexity part of the subarachnoid space were also sufficiently high for DESH detection. In particular, the optimal thresholds of the volumes (volume ratios) of the ventricle and high-convexity part of the subarachnoid space were set at 100 mL (6%) and 30 mL (2%), respectively. The AUCs of the volumes and volume ratios of the total ventricles for ventriculomegaly detection were over 0.98, which was higher than that of the Venthi index (<0.95), whereas that of the DESH index for THC detection was 0.99 and higher than those of the volumes and volume ratios of the high-convexity part of the subarachnoid space (0.96 and 0.97, respectively), as shown in Table 4. Furthermore, the volume and volume ratio of the Sylvian fissure and basal cistern had relatively lower AUCs (0.91 and 0.86, respectively).

**Table 3.** Diagnostic performance of the deep-learning algorithm for detecting disproportionately enlarged subarachnoid space hydrocephalus (DESH) on MRI.

	AUC (95%CIs)	Sens (95%CIs)	Spec (95%CIs)	Threshold	Total (1009)	DESH (101)	non-DESH (908)	OR	95% CIs
DESH index	0.99 (0.99-1.00)	96.0 (92.1-99.0)	96.1 (94.9-97.4)	6	131/878	96/5	35/873	468	178-1600
Venthi index	0.99 (0.98-1.00)	95.0 (91.1-99.0)	96.8 (95.5-96.8)	3.6	125/884	96/5	29/879	566	213-1865
Sylhi index	0.98 (0.97-0.99)	95.0 (91.1-99.0)	91.5 (89.8-93.3)	2.2	173/836	96/5	77/831	205	81-657
Ventricle volume (mL)	0.95 (0.93-0.97)	85.1 (78.2-92.1)	95.0 (93.5-96.4)	100	137/872	86/15	51/857	95	50-190
Ventricle volume ratio (%)	0.95 (0.93-0.97)	92.1 (86.1-97.0)	87.3 (85.2-89.4)	6	199/810	90/11	109/799	60	31-127
HCS volume (mL)	0.95 (0.93-0.98)	93.1 (87.9-97.0)	85.6 (83.3-87.9)	30	243/766	95/6	148/760	81	35-231
HCS volume ratio (%)	0.96 (0.94-0.98)	93.1 (88.1-97.0)	91.0 (89.1-92.8)	2	201/808	94/7	107/801	100	45-261
Syl+BC volume (mL)	0.70 (0.64-0.76)	48.5 (38.6-58.4)	87.4 (85.4-89.5)	73	166/843	49/52	117/791	6.4	4.0-10.1
Syl+BC volume ratio (%)	0.67 (0.61-0.73)	47.5 (38.6-57.4)	83.8 (81.3-86.1)	5	178/831	45/56	133/775	4.7	3.0-7.4

DESH index = total ventricular volume and Sylvian fissure and basal cistern volume divided by the high-convexity part of the subarachnoid space volume; Venthi index = ventricular volume divided by the high-convexity part of the subarachnoid space volume; Sylhi index = Sylvian fissure and basal cistern volume divided by the high-convexity part of the subarachnoid space volume

Abbreviations: 95% CIs = 95% confidential intervals; HCS = high-convexity part of the subarachnoid space; OR = odds ratio; Sens = sensitivity; Spec = specificity; Syl + BC, Sylvian fissure and basal cistern.

**Table 4.** Diagnostic performance of the deep-learning algorithm for detecting ventriculomegaly, tightened sulci in the high convexities, and Sylvian fissure dilation on MRI.

	AUC	Sens	Spec	Threshold	Total (1009)	Positive	Negative	OD	95% CIs
**For ventriculomegaly**						195	814		
Venthi index	0.94 (0.93-0.96)	93.3 (89.7-96.9)	81.1 (78.3-83.8)	1.8	342/667	182/13	160/654	57	31-112
Ventricle volume (mL)	0.99 (0.98-1.00)	95.9 (92.8-98.5)	94.1 (92.4-95.6)	81.5	234/775	186/9	48/766	364	155-767
Ventricle volume ratio (%)	0.98 (0.97-0.99)	92.3 (88.2-95.9)	95.2 (93.6-96.6)	5.9	213/796	176/19	37/777	191	106-361
**For tightened sulci in the high convexities**						93	916		
DESH index	0.99 (0.99-1.00)	95.7 (90.3-98.9)	96.9 (95.9-97.9)	6.5	116/893	88/5	28/888	543	203-1957
HCS volume (mL)	0.96 (0.93-0.98)	87.1 (79.6-93.5)	94.4 (92.9-95.9)	23.6	134/875	81/12	53/863	109	55-233
HCS volume ratio (%)	0.97 (0.95-0.99)	94.6 (89.2-98.9)	90.8 (89.0-92.7)	1.9	178/831	88/5	90/826	160	64-511
**For Sylvian fissure dilation**						241	768		
Sylhi index	0.81 (0.78-0.84)	73.0 (67.6-78.8)	73.4 (70.4-76.3)	1.5	407/601	181/60	226/542	7.2	5.1-10.2
Syl + BC volume (mL)	0.91 (0.88-0.93)	90.0 (86.3-93.8)	77.0 (74.0-79.7)	60.0	394/615	217/24	177/591	30	19-50
Syl + BC volume ratio (%)	0.86 (0.83-0.89)	75.5 (69.7-80.9)	80.2 (77.5-82.9)	4.5	319/690	177/64	142/626	12	8.6-17.4

DESH index = total ventricular volume and Sylvian fissure and basal cistern volume divided by the high-convexity part of the subarachnoid space volume; Venthi index = ventricular volume divided by the high-convexity part of the subarachnoid space volume; Sylhi index = Sylvian fissure and basal cistern volume divided by the high-convexity part of the subarachnoid space volume

Abbreviations: 95% CIs = 95% confidential intervals; HCS = high-convexity part of the subarachnoid space; OR = odds ratio; Sens = sensitivity; spec = specificity; Syl + BC = Sylvian fissure and basal cistern.

## Discussion

In this study, DESH was identified in all patients with Hakim’s disease (iNPH) and those with Hakim's disease and Alzheimer’s disease comorbidity, although the severity of DESH varied. The DESH index is utilized as a quantitative measure for assessing DESH. A cutoff value exceeding 6 suggests a high probability of DESH, with higher index values corresponding to increased severity of DESH. Moreover, we found that the DESH evaluation was highly dependent on THC evaluation, while the DESH index was also dependent on the high-convexity part of the subarachnoid space volume (Figure 3). Gunter et al.^23^ also reported that THC was the most important radiologic marker for DESH determination that did not overlap with any common neurodegenerative disorders, unlike ventriculomegaly or SFD. Our conclusions from this study are also consistent with their findings.

In addition to the DESH index, the Venthi index measures the impact of ventriculomegaly in DESH, while the Sylhi index assesses the influence of SFD in DESH. These indices may facilitate the examination of the relationship between the DESH pattern and gait disturbances, as well as cognitive impairment in Hakim’s disease, and could pave the way for prospective studies to quantitatively predict symptom improvement in the future. As Hakim’s disease becomes more prevalent with age and can even be detected in individuals as old as 90, early identification of patients is crucial, although surgical intervention may not always be appropriate. Current international and Japanese guidelines include a response to the tap test (probable iNPH) and symptom improvement after shunt surgery (definite iNPH) as part of the diagnostic criteria.^1^^,^^2^ However, the high false-negative rate of the CSF tap test remains a challenge,^1^^,^^2^ and shunt surgery carries risks such as shunt malfunction and infection. In such cases, even if reoperations are performed, symptom improvement may not be achieved, potentially leading to the misunderstanding that the diagnosis of iNPH was incorrect. The diagnosis of Hakim’s disease can be made more objectively without relying on the tap test or shunt surgery by evaluating both the patient’s symptoms and DESH findings on imaging, allowing therapeutic interventions to be tailored to each individual patient’s condition. Furthermore, by using this app, it has become possible to quantify not only the differentiation between Hakim’s disease and Alzheimer’s disease but also the potential for their coexistence. The co-occurrence of Alzheimer’s disease may reduce the degree and duration of shunt effectiveness in patients with Hakim’s disease, making it essential for determining treatment eligibility. Therefore, this app not only aids in identifying previously overlooked patients but also predicts which symptoms are more likely to improve based on DESH-related indices and specific morphological characteristics, potentially guiding the selection of appropriate therapeutic interventions and offering a more tailored approach to treatment.

This study has several limitations. First, the comparison of the proposed 3D indices and extracted volumes and volume ratios with conventional 2D indices, such as Evans index, Z-Evans index,^32^ brain per ventricle ratio,^33^ and callosal angle, was not performed because the measurement of 2D indices is also time-consuming. Second, of the 101 participants determined with DESH, 77 (76%) were diagnosed with Hakim’s disease, while the others were determined to present with DESH on MRI. However, among the others with DESH, the possibility that true Hakim’s disease may be hidden or that Hakim’s disease coexisted with other diseases cannot be ruled out. Third, all MRI data were subjectively evaluated for the presence or absence of DESH, including ventriculomegaly, SFD, and THC. Although subjective evaluation can be ambiguous and often results in differing judgments among experts, it was unavoidable for the development of objective evaluation methods. Fourth, the reliability and validity of the ROI volumes automatically extracted by these applications have not yet been fully validated in this study. Finally, we did not investigate the relationship between the DESH index and the various symptoms of Hakim’s disease. The relationship of the DESH index to the severity of symptoms of Hakim’s disease and its impact on symptom improvement after shunt surgery should be examined in the future.

## Conclusions

This study provided evidence confirming that a deep learning-based application could quantitatively and accurately evaluate DESH, which is the key imaging marker for Hakim’s disease (iNPH) using 3D T1-weighted MRI. By utilizing deep learning-based automatic segmentation technology, we expect to improve the diagnostic accuracy of Hakim’s disease (iNPH) by eliminating the ambiguity associated with DESH evaluation—an issue that has persisted with conventional subjective assessments, even when using 2D indices including Evans index and callosal angle, which have proven insufficient. Furthermore, this technology would be expected to provide patients with Hakim’s disease (iNPH) a better chance of receiving appropriate treatment at the optimal time.

## Supplementary Material

umae027_Supplementary_Data

## Data Availability

The MRI data in this study are not available to the community via any open repositories, because the ethics committees have approved the sharing of the MRI data in this research with collaborative institutes and does not allow its being provided to other institutions. The data will be available only on the condition that the ethics committees approve any new participation in the collaborative research. Sharing the algorithm codes for these 2 applications is not possible as they are commercialized as medical devices by FUJIFILM Corporation.
