# Supplementary material for: Deep learning assessment of disproportionately enlarged subarachnoid-space hydrocephalus in Hakim’s disease or idiopathic normal pressure hydrocephalus
Source: Radiol Adv. 2024 Nov 4;1(3):umae027. doi: 10.1093/radadv/umae027 (PMC12429205; doi:10.1093/radadv/umae027)
Supplement: umae027_Supplementary_Data [file umae027_Supplementary_Data.zip › Table S1.pdf]

Title: Deep Learning Assessment of Disproportionately Enlarged Subarachnoid-Space Hydrocephalus in Hakim's Disease or Idiopathic Normal Pressure Hydrocephalus

Table S1:

Comparison between disproportionately enlarged subarachnoid space hydrocephalus (DESH) and non-DESH group on brain MRI

	Total (n = 1009)		DESH (n = 101)		Non-DESH (n = 908)		*P*
	Mean ± SD	Range	Mean ± SD	Range	Mean ± SD	Range	
Age	73.8 ± 13.6	21–99	77.8 ± 7.1	57–96	73.4 ± 14.0	21–99	0.03
MMSE	22.6 ± 6.0	0–30	22.3 ± 6.0	1–30	22.7 ± 6.0	0–30	0.44
Total ventricle volume (mL)	64.3 ± 32.9	9.4–222.3	125.2 ± 33.9	53.6–222.3	57.5 ± 24.9	9.4–152.4	<0.001
Total ventricle volume ratio (%)	4.5 ± 2.1	0.7–12.8	8.4 ± 1.9	3.7–12.8	4.0 ± 1.7	0.7–9.7	<0.001
HCS volume (mL)	41.1 ± 15.8	2.9–86.9	16.6 ± 9.9	2.9–64.9	43.8 ± 13.9	12.9–86.9	<0.001
HCS volume ratio (%)	2.9 ± 1.1	0.2–5.7	1.1 ± 0.6	0.2–3.6	3.1 ± 0.9	1.0–5.7	<0.001
Syl+BC volume (mL)	58.4 ± 16.4	26.7–145.9	71.2 ± 21.3	36.9–133.3	57.0 ± 15.1	26.7–145.9	<0.001
Syl+BC volume ratio (%)	4.1 ± 1.1	1.9–10.0	4.8 ± 1.4	2.5–8.8	4.0 ± 1.0	1.9–10.0	<0.001
CSF	352 ± 81.7	167.3–668.5	350.1 ± 67.2	244–626.2	352.3 ± 83.2	167.3–668.5	0.93
DESH index	4.4 ± 6.6	0.8–93.5	17.7 ± 15.0	4.2–93.5	2.9 ± 1.4	0.8–9.5	<0.001
Venthi index	2.5 ± 4.9	0.2–70.2	11.9 ± 11.6	1.8–70.2	1.5 ± 0.9	0.2–5.9	<0.002
Sylhi index	1.9 ± 1.8	0.6–23.4	5.8 ± 3.7	1.6–23.4	1.4 ± 0.6	0.6–4.7	<0.003

P, probability values of the mean values that were calculated by using the Mann–Whitney–Wilcoxon test; HCS, high-convexity part of the subarachnoid space; Syl+BC, Sylvian fissure and basal cistern; CSF, cerebrospinal fluid; DESH index = (total ventricular volume) + (Sylvian fissure and basal cistern volume) / (high-convexity part of the subarachnoid space volume); Venthi index = (ventricular volume) (high-convexity part of the subarachnoid space volume); Sylhi index = (Sylvian fissure and basal cistern volume) (high-convexity part of the subarachnoid space volume)
