# Supplementary material for: Deep learning assessment of disproportionately enlarged subarachnoid-space hydrocephalus in Hakim’s disease or idiopathic normal pressure hydrocephalus
Source: Radiol Adv. 2024 Nov 4;1(3):umae027. doi: 10.1093/radadv/umae027 (PMC12429205; doi:10.1093/radadv/umae027)
Supplement: umae027_Supplementary_Data [file umae027_Supplementary_Data.zip › STARD-2015-Checklist-RSNA.pdf]

STARD 2015 Checklist

Section & Topic	No.	Item	✔ (N/A)
**TITLE OR ABSTRACT**			
	1	Identification as a study of diagnostic accuracy using at least one measure of accuracy (such as sensitivity, specificity, predictive values, or AUC)	✔
**ABSTRACT**			
	2	Structured summary of study design, methods, results, and conclusions (for specific guidance, see STARD for Abstracts)	✔
**INTRODUCTION**			
	3	Scientific & clinical background, including the intended use and clinical role of the index test	✔
	4	Study objectives and hypotheses	✔
**METHODS**			
Study design	5	Whether data collection was planned before the index test and reference standard were performed (prospective study) or after (retrospective study)	✔
Participants	6	Eligibility criteria	✔
	7	On what basis potentially eligible participants were identified (such as symptoms, results from previous tests, inclusion in registry)	✔
	8	Where and when potentially eligible participants were identified (setting, location and dates)	✔
	9	Whether participants formed a consecutive, random or convenience series	✔
Test methods	10a	Index test, in sufficient detail to allow replication	✔
	10b	Reference standard, in sufficient detail to allow replication	✔
	11	Rationale for choosing the reference standard (if alternatives exist)	N/A
	12a	Definition of and rationale for test positivity cut-offs or result categories of the index test, distinguishing pre-specified from exploratory	✔
	12b	Definition of and rationale for test positivity cut-offs or result categories of the reference standard, distinguishing pre-specified from exploratory	✔
	13a	Whether clinical information and reference standard results were available to the performers/readers of the index test	✔
	13b	Whether clinical information and index test results were available to the assessors of the reference standard	No
Analysis	14	Methods for estimating or comparing measures of diagnostic accuracy	✔
	15	How indeterminate index test or reference standard results were handled	✔
	16	How missing data on the index test and reference standard were handled	✔
	17	Any analyses of variability in diagnostic accuracy, distinguishing pre-specified from exploratory	✔
	18	Intended sample size and how it was determined	✔
**RESULTS**			
Participants	19	Flow of participants, using a diagram. Include the figure number (preferably figure 1) or page number	No
	20	Baseline demographic and clinical characteristics of participants	✔
	21a	Distribution of severity of disease in those with the target condition	No
	21b	Distribution of alternative diagnoses in those without the target condition	No
	22	Time interval and any clinical interventions between index test and reference standard	No
Test results	23	Cross tabulation of the index test results (or their distribution) by the results of the reference standard	✔
	24	Estimates of diagnostic accuracy and their precision (such as 95% confidence intervals)	No
	25	Any adverse events from performing the index test or the reference standard	N/A
**DISCUSSION**			
	26	Study limitations, including sources of potential bias, statistical uncertainty, and generalisability	✔
	27	Implications for practice, including the intended use and clinical role of the index test	✔
**OTHER INFORMATION**			
	28	Registration number and name of registry	✔
	29	Where the full study protocol can be accessed	✔
	30	Sources of funding and other support; role of funders	✔

*N/A stands for not applicable and may be a reasonable choice depending on the type of study performed

RSNA®
