# Supplementary material for: Deep learning assessment of disproportionately enlarged subarachnoid-space hydrocephalus in Hakim’s disease or idiopathic normal pressure hydrocephalus
Source: Radiol Adv. 2024 Nov 4;1(3):umae027. doi: 10.1093/radadv/umae027 (PMC12429205; doi:10.1093/radadv/umae027)
Supplement: umae027_Supplementary_Data [file umae027_Supplementary_Data.zip › Table S1.docx]

**Table S1:**

**Comparison between disproportionately enlarged subarachnoid space hydrocephalus (DESH) and non-DESH group on brain MRI**

|  | Total (n = 1009) | | DESH (n = 101) | | Non-DESH (n = 908) | | *P* |
| --- | --- | --- | --- | --- | --- | --- | --- |
|  | Mean ± SD | Range | Mean ± SD | Range | Mean ± SD | Range |  |
| Age | 73.8 ± 13.6 | 21–99 | 77.8 ± 7.1 | 57–96 | 73.4 ± 14.0 | 21–99 | 0.03 |
| MMSE | 22.6 ± 6.0 | 0–30 | 22.3 ± 6.0 | 1–30 | 22.7 ± 6.0 | 0–30 | 0.44 |
| Total ventricle volume (mL) | 64.3 ± 32.9 | 9.4–222.3 | 125.2 ± 33.9 | 53.6–222.3 | 57.5 ± 24.9 | 9.4–152.4 | <0.001 |
| Total ventricle volume ratio (%) | 4.5 ± 2.1 | 0.7–12.8 | 8.4 ± 1.9 | 3.7–12.8 | 4.0 ± 1.7 | 0.7–9.7 | <0.001 |
| HCS volume (mL) | 41.1 ± 15.8 | 2.9–86.9 | 16.6 ± 9.9 | 2.9–64.9 | 43.8 ± 13.9 | 12.9–86.9 | <0.001 |
| HCS volume ratio (%) | 2.9 ± 1.1 | 0.2–5.7 | 1.1 ± 0.6 | 0.2–3.6 | 3.1 ± 0.9 | 1.0–5.7 | <0.001 |
| Syl+BC volume (mL) | 58.4 ± 16.4 | 26.7–145.9 | 71.2 ± 21.3 | 36.9–133.3 | 57.0 ± 15.1 | 26.7–145.9 | <0.001 |
| Syl+BC volume ratio (%) | 4.1 ± 1.1 | 1.9–10.0 | 4.8 ± 1.4 | 2.5–8.8 | 4.0 ± 1.0 | 1.9–10.0 | <0.001 |
| CSF | 352 ± 81.7 | 167.3–668.5 | 350.1 ± 67.2 | 244–626.2 | 352.3 ± 83.2 | 167.3–668.5 | 0.93 |
| DESH index | 4.4 ± 6.6 | 0.8–93.5 | 17.7 ± 15.0 | 4.2–93.5 | 2.9 ± 1.4 | 0.8–9.5 | <0.001 |
| Venthi index | 2.5 ± 4.9 | 0.2–70.2 | 11.9 ± 11.6 | 1.8–70.2 | 1.5 ± 0.9 | 0.2–5.9 | <0.002 |
| Sylhi index | 1.9 ± 1.8 | 0.6–23.4 | 5.8 ± 3.7 | 1.6–23.4 | 1.4 ± 0.6 | 0.6–4.7 | <0.003 |

*P*, probability values of the mean values that were calculated by using the Mann–Whitney–Wilcoxon test; HCS, high-convexity part of the subarachnoid space; Syl+BC, Sylvian fissure and basal cistern; CSF, cerebrospinal fluid; DESH index = (total ventricular volume) + (Sylvian fissure and basal cistern volume) / (high-convexity part of the subarachnoid space volume); Venthi index = (ventricular volume) (high-convexity part of the subarachnoid space volume); Sylhi index = (Sylvian fissure and basal cistern volume) (high-convexity part of the subarachnoid space volume)
